# Low handgrip strength, GLIM-defined malnutrition, or their coexistence: which has the greatest impact on health-related quality of life in patients with cancer?

**DOI:** 10.1186/s12885-026-15823-8

**Published:** 2026-03-21

**Authors:** Afonso Marcos Alves de Souza, Maria Claudia Bernardes Spexoto

**Affiliations:** Faculty of Health Sciences (FCS), Federal University of Grande Dourados (UFGD), Graduate Program in Food, Nutrition and Health (PPGANS), Dourados, Brazil

**Keywords:** Nutritional assessment, Neoplasms, Malnutrition, Dynamometry, Health-related quality of life

## Abstract

**Background:**

Malnutrition and reduced muscle strength are prevalent among patients with cancer and are both linked to impaired health-related quality of life (HRQoL). Although the individual impacts of these conditions are well documented, their combined effect remains insufficiently explored. This study aimed to examine how coexisting GLIM-defined malnutrition and low handgrip strength (HGS) affect HRQoL.

**Methods:**

This-cross-sectional study (November 2024-June 2025) involved adult patients with cancer undergoing clinical treatment. Sociodemographic, clinical, lifestyle, and anthropometric information was collected. Malnutrition was defined according to the Global Leadership Initiative on Malnutrition (GLIM) criteria, and low HGS was used as a marker of reduced muscle strength. Coexistence was characterized by the simultaneous presence of malnutrition and low HGS. HRQoL was evaluated using the EORTC QLQ-C30 (0–100 scores), where higher functional scores indicate better HRQoL and higher symptom scores indicate greater symptom burden. Continuous variables were summarized as medians and interquartile ranges, and group differences were tested with nonparametric methods. Adjusted associations were examined using univariate general linear models, expressed as adjusted mean differences (Δ) and effect sizes (partial eta-squared, ${\eta }_{p}^{2}$). Statistical significance was set at *p* < 0.05.

**Results:**

After adjustment, low HGS showed significant and stronger associations with all functional scales and most symptom scales, whereas GLIM-defined malnutrition alone remained associated only with appetite loss. The coexistence of malnutrition and low HGS was associated with poorer physical (Δ = -35.1; *p* = 0.002; ${\eta }_{p}^{2}$ = 0.240), role (Δ = -40.3; *p* = 0.014; ${\eta }_{p}^{2}$= 0.216), and emotional functioning (Δ = -27.7; *p* = 0.044; ${\eta }_{p}^{2}$ = 0.125). Coexistence was also associated with higher levels of fatigue (Δ = 37.9; *p* < 0.01; ${\eta }_{p}^{2}$= 0.201), nausea and vomiting (Δ = 29.5; *p* ≤ 0.020; ${\eta }_{p}^{2}$= 0.209), pain (Δ = 36.0; *p* = 0.027; ${\eta }_{p}^{2}$= 0.134), dyspnea (Δ = 18.0; *p* = 0.042; ${\eta }_{p}^{2}$= 0.132), and appetite loss (Δ = 39.6; *p* = 0.015; ${\eta }_{p}^{2}$= 0.132).

**Conclusions:**

The coexistence of GLIM-defined malnutrition and low HGS is associated with poorer HRQoL in patients with cancer, with low handgrip strength being particularly relevant to HRQoL impairment.

**Supplementary Information:**

The online version contains supplementary material available at 10.1186/s12885-026-15823-8.

## Introduction

Malnutrition is highly prevalent among patients with cancer and varies widely by cancer type and stage; it results from a complex interplay of tumor-related effects, systemic inflammation, antineoplastic therapies, and reduced food intake [1]. This process often leads to loss of muscle mass and strength, thereby compromising treatment response, survival, and health-related quality of life (HRQoL) [2, 3].

To standardize the diagnosis of malnutrition, the Global Leadership Initiative on Malnutrition (GLIM) proposed diagnostic criteria that require the combination of at least one phenotypic criterion (non-volitional weight loss, low body mass index, or reduced muscle mass) and one etiologic criterion (reduced food intake or assimilation and disease burden/inflammation) [4]. In parallel, handgrip strength (HGS) has been recognized as a functional marker that is independent of muscle mass and associated with adverse clinical outcomes, including poorer HRQoL in patients with cancer [5, 6]. HRQoL is a key clinical outcome in oncology practice, capturing functional limitations, symptom burden, and overall well-being. The European Organisation for Research and Treatment of Cancer Quality of Life Questionnaire Core 30 (EORTC QLQ-C30) is widely used for this purpose, allowing assessment of physical, emotional, and social functioning, as well as disease- and treatment-related symptoms [7].

Despite the well-established clinical and prognostic relevance of malnutrition and reduced muscle strength when examined separately, evidence on the impact of these conditions on HRQoL in patients with cancer remains limited, and further research exploring functional measures such as handgrip strength in relation to patient-reported outcomes has been encouraged [8]. Previous studies, such as that by Landgrebe et al*.* [9], showed that malnutrition defined by the GLIM criteria was associated with poorer HRQoL (HR = 2.1; 95% CI 1.2–3.6). In contrast, Silva et al*.* (r = 0.26; 95% CI 0.07–0.35) [10] and Caroline et al*.* (R^2^ = 0.195; *p* < 0.01) [11] found that reduced muscle strength, assessed by HGS, was also related to poorer HRQoL. However, to date, no studies have evaluated the impact of coexisting these two conditions on HRQoL in this population or their effects on the different functional and symptom scales of the EORTC QLQ-C30. Therefore, this study aimed to investigate the impact of coexisting GLIM-defined malnutrition and low handgrip strength on global HRQoL and its scales in patients with cancer. We hypothesized that this coexistence would exert an additive effect, resulting in more severely impaired HRQoL than either condition alone.

## Methods

### Study population

All adult patients with a medical diagnosis of cancer who were receiving care at a hospital specializing in oncology in the state of Mato Grosso do Sul, Brazil, were considered for inclusion. Eligible patients were men or women aged 20 years or older, regardless of literacy status, with sufficient cognitive and physical capacity to understand and complete the physical tests and questionnaires (study protocol), a time since diagnosis of up to 12 months, and the ability to walk, even when using assistive devices (cane, walker, or similar). Exclusion criteria were complete inability to ambulate without assistance, neuroatypical status identified in the medical records, pregnancy, edema in the lower limbs or any restriction that precluded handgrip strength assessment, and non-melanoma skin cancer. A previously trained team collected the data using a structured questionnaire.

### Sample size and power analysis

All eligible patients during the data collection period were invited to participate, yielding a final sample of 130. No a priori sample size calculation was performed, which we acknowledge as a limitation of this observational design. To assess whether the sample size was adequate, we performed a sensitivity analysis based on the observed effect sizes (partial eta-squared,${\eta }_{p}^{2}$) for the main adjusted outcomes from the EORTC QLQ-C30. In the adjusted models, effect sizes ranged from moderate to large ((${\eta }_{p}^{2}$= 0.132–0.240; f = 0.39–0.56, according to the conversion proposed by Cohen [12]: f = √((${\eta }_{p}^{2}$/(1 − (${\eta }_{p}^{2}$)). These values indicate that, assuming α = 0.05 and power = 0.80, the sample of 130 participants was sufficient to detect the observed effects in the main EORTC QLQ-C30 scales (physical, role, and emotional functioning and the symptom scales for fatigue, nausea and vomiting, pain, dyspnea, appetite loss, and diarrhea).

### Independent variables (exposures)

#### Muscle strength

Handgrip strength (HGS, kg) was measured using a SAEHAN® hydraulic hand dynamometer (model SH5001). Patients were first familiarized with the device and then assessed in the seated position, with both arms flexed at the elbow to 90°. They were instructed to hold the dynamometer and squeeze it with maximal effort. Measurements were obtained three times per hand at 1-min intervals, and the highest value was used in the analyses. Low handgrip strength was defined using sex-specific cut-off points of < 32 kg in men and < 21 kg in women [13]. Although the 2019 consensus of the European Working Group on Sarcopenia in Older People (EWGSOP2) [14] recommends different cut-off values for older European adults (< 27 kg for men and < 16 kg for women), the guideline also recognizes that handgrip strength cut-off points may vary across populations due to differences in anthropometry and body composition. Therefore, we adopted cut-off points previously validated in Brazilian adults, which may provide a more appropriate classification of low muscle strength in our study context.

#### Malnutrition

Malnutrition was diagnosed according to the Global Leadership Initiative on Malnutrition (GLIM) criteria, without prior nutritional risk screening tool [4]. We applied the GLIM criteria directly because all patients had a confirmed cancer diagnosis and were being followed at a high-complexity tertiary cancer center, which characterizes a nutritionally at-risk population. This approach has been used in previous oncology studies and is therefore methodologically justified [15, 16].

To diagnose GLIM-defined malnutrition, at least one phenotypic and one etiologic criterion had to be present. Reduced muscle mass was used as the phenotypic criterion. Age-specific anthropometric indicators were applied to assess muscle mass, as calf circumference is widely recommended and validated as a surrogate marker of low muscle mass in older adults, whereas arm muscle circumference provides an appropriate estimate of peripheral muscle reserves in younger individuals [17–21]. In participants aged ≥ 60 years, muscle mass was assessed using calf circumference (CC), measured with a flexible, non-stretch tape in accordance with Lohman’s recommendations [22]. Values ≤ 34 cm in men and ≤ 33 cm in women were considered indicative of reduced muscle mass [23]. In participants aged < 60 years, arm muscle circumference (AMC) was calculated from mid-arm circumference (MAC) and triceps skinfold thickness (TSF), and values < 90% of the 50th percentile for sex and age were classified as indicating malnutrition [24, 25]. The etiologic criterion was the presence of acute or chronic disease with an associated inflammatory response, namely cancer, which was present in all participants.

Malnutrition severity was determined based on phenotypic criteria and categorized as moderate or severe. Moderate malnutrition was defined as 5–10% weight loss in the previous 6 months, 10–20% weight loss over more than 6 months, BMI < 20 kg/m^2^ in individuals < 70 years or < 22 kg/m^2^ in those ≥ 70 years, or a mild-to-moderate deficit in fat-free mass. Severe malnutrition was defined as > 10% weight loss in the previous 6 months, > 20% weight loss over more than 6 months, BMI < 18.5 kg/m^2^ in individuals < 70 years or < 20 kg/m^2^ in those ≥ 70 years, or a severe deficit in fat-free mass.

#### Coexistence

Coexistence was defined as simultaneously meeting the GLIM criteria for malnutrition and the study definition of low handgrip strength.

### Dependent variable (outcome)

#### Health-related quality of life and its scales

Health-related quality of life (HRQoL) was assessed with the European Organisation for Research and Treatment of Cancer Quality of Life Questionnaire Core 30, version 3.0 (EORTC QLQ-C30), using the Portuguese version provided by the European Organisation for Research and Treatment of Cancer (qol.eortc.org), which comprises 30 items organized into two Sects [26].

The first section includes 28 items rated on a 4-point Likert scale, with response options with response options 1 (“Not at all”), 2 (“A little”), 3 (“Quite a bit”), and 4 (“Very much”). This section covers aspects of functioning, including physical effort, walking short and long distances, mobility, and the performance of daily activities such as dressing, getting up, and using the bathroom. It also addresses the patient’s experience during the previous week in terms of difficulties with everyday and leisure activities; dyspnea; pain; sleep disturbances; gastrointestinal symptoms (nausea, vomiting, and diarrhea); fatigue; psychological symptoms such as worry, nervousness, irritability, and problems with concentration and memory; and the impact of the disease or its treatment on family life, social activities, and financial situation.

The second section comprises two items rated on a 7-point Likert scale ranging from 1 (“very poor”) to 7 (“excellent”). The first item asks patients to rate their overall health during the previous week, and the second asks them to rate their overall quality of life in the same period. The questionnaire was administered in a single session, and all responses referred to the previous week. Although it is designed as a self-administered and self-explanatory instrument, it was interviewer-administered in this study to minimize data collection errors.

Scores were calculated according to the EORTC QLQ-C30 Scoring Manual, which includes 15 scales: physical functioning (5 items: 1–5), role functioning (2 items: 6 and 7), emotional functioning (4 items: 21–24), cognitive functioning (2 items: 20 and 25), social functioning (2 items: 26 and 27), fatigue (3 items: 10, 12, and 18), nausea and vomiting (2 items: 14 and 15), pain (2 items: 9 and 19), dyspnea (1 item: 8), insomnia (1 item: 11), appetite loss (1 item: 13), constipation (1 item: 16), diarrhea (1 item: 17), and financial difficulties (1 item: 28), as well as the global health status/QoL scale derived from items 29 and 30. For each scale, a raw score (RS) was obtained by averaging the corresponding items, as shown in Eq. 1:

Computation of EORTC QLQ-C30 raw scores.

1 $$RS =\frac{\left(I1 + I2 + \dots + In\right)}{n}$$

After computing the raw scores, all scales were linearly transformed to a 0–100 scale. For the functional scales and the global health status/QoL scale, higher scores indicate better HRQoL, whereas for the symptom scales, higher scores indicate greater symptom burden.

#### Covariables

Sociodemographic covariates included age (years); marital status (never married, married, widowed, or separated/divorced); current employment (yes/no); self-reported race/skin color (White, Black, Brown [pardo], and Yellow), which was later collapsed into White versus non-White for analysis; educational attainment (< 4 years, 4–8 years, > 8–11 years, and > 11 years of schooling); and economic class, classified according to the Brazilian Economic Classification Criterion 2022 (Critério de Classificação Econômica Brasil 2022) [27].

Lifestyle covariates included alcohol consumption (never drank, former drinker, current drinker); smoking status (never smoked, former smoker, current smoker); and physical activity level, assessed with the International Physical Activity Questionnaire (IPAQ), short form, for the Brazilian population [28]. Physical activity level was categorized according to World Health Organization guidelines on physical activity and sedentary behavior as sufficient for patients who reported 150–300 min/week of moderate-intensity physical activity or 75–150 min/week of vigorous-intensity physical activity, and insufficient for those who did not meet these criteria [29].

Clinical covariates included tumor stage (0, I, II, or III); presence or absence of metastasis; type of cancer treatment (chemotherapy, radiotherapy, hormone therapy, or a combination of two or more modalities); tumor diagnosis and site, classified as breast, prostate, digestive system, or other; and the presence or absence of pre-existing chronic diseases.

Body mass index (BMI, kg/m^2^) was calculated and classified according to World Health Organization criteria [30]. Calf circumference values were interpreted using BMI-adjusted cut-off points proposed by Gonzalez et al*.* [31].

### Data processing and statistical analysis

All statistical analyses were performed using SPSS, version 22.0 (IBM Corp., Armonk, NY, USA). The normality of continuous variables was assessed with the Shapiro–Wilk test and by visual inspection of histograms. Because EORTC QLQ-C30 scale scores did not follow a normal distribution, we summarized them as medians and interquartile ranges (IQR). Categorical variables were described as absolute and relative frequencies.

As a first step, we compared global HRQoL and QLQ-C30 scale scores between groups using nonparametric Mann–Whitney tests (two groups) or Kruskal–Wallis tests (more than two groups). For pairwise contrasts following multiple comparisons, we reported the U statistic, standardized Z value, *p*-value, and effect size (r), calculated as the Z value divided by the square root of N (r = Z/√N). Effect sizes were interpreted according to conventional thresholds: small (r close to 0.10), medium (r close to 0.30), and large (r ≥ 0.50).

Next, to explore adjusted associations between the exposures and the EORTC QLQ-C30 scale scores, we fitted univariate general linear models (GLM-Univariate), assuming normally distributed residuals. Exposure variables included GLIM-defined malnutrition (no = 0/yes = 1), handgrip strength (adequate = 0/low handgrip strength = 1), and a four-category coexistence variable coded as 1 = neither condition, 2 = GLIM-defined malnutrition only, 3 = low handgrip strength only, and 4 = coexistence of both. Models were adjusted for sex, age, BMI, tumor site, and clinical stage. Missing data were limited and are reported in Table 1. All adjusted models were fitted using complete-case analysis. Covariates were prespecified a priori to represent major demographic and clinical confounders while maintaining model parsimony and statistical precision given the sample size. Additional variables collected (e.g., physical activity, comorbidities, treatment modality, smoking and alcohol use) were not included in the main adjusted models to reduce the risk of overfitting, but were considered when interpreting the potential for residual confounding.

**Table 1:** Sociodemographic, lifestyle and clinical characteristics

Variables	n	%
*Sociodemographic characteristics*		
Age (years) (mean ± SD)	59.5 ± 11.6	
Age group		
Adult	66	50.8
Older adult	64	49.2
Sex		
Male	34	26.2
Female	96	73.8
Self-reported race/skin color		
White or Yellow	53	40.8
Black or Brown	76	58.2
Marital status		
Married	73	56.2
Never married	27	20.8
Widowed	18	13.8
Separated/Divorced	11	8.5
Educational attainment		
< 4 years	17	13.2
4–8 years	27	20.9
> 11 years	61	47.3
Illiterate	24	18.6
Economic class		
A	4	3.1
B	47	36.2
C	64	49.2
D e E	15	11.5
*Lifestyle*		
Alcohol consumption		
Never drank	63	48.5
Former drinker	57	43.8
Current drinker	10	7.7
Smoking status		
Never smoked	89	68.5
Former smoker	34	26.2
Current smoker	7	5.4
Physical activity level		
Insufficient	117	90.0
Sufficient	13	10.0
Clinical characteristics		
Cancer site		
Breast	69	53.1
Prostate	15	11.5
Digestive system	24	18.5
Other	22	16.9
Clinical stage^a^		
I	10	8.5
II	33	28.0
III	47	39.8
IV	28	23.7
Metastasis^b^		
No	59	49.2
Yes	61	50.8
Chronic diseases		
No	82	63.1
Yes	48	36.9
Treatment		
Chemotherapy	87	71.9
Radiotherapy	4	3.3
Hormone therapy	6	5.0
Two or more treatment modalities	24	19.8
BMI (mean ± SD)^c^	27.2 ± 5.8	
HGS (mean ± SD)	25.3 ± 10.9	
Adequate (men ≥ 32 kg; women ≥ 21 kg)	79	60.8
Low muscle strength (men < 32 kg; women < 21 kg)	51	39.2
CC (mean ± SD)^d^	35.5 ± 4.5	
Adequate	88	68.2
Low muscle mass	41	31.8
BMI-adjusted CC (mean ± SD)	32.2 ± 4.5	
Adequate	43	33.1
Low muscle mass	87	66.9

*BMI* Body mass index, *HGS* Handgrip strength, *CC * Calf circumference, *GLIM *Global Leadership Initiative on Malnutrition, *SD *Standard deviation

Missing data: ^a^n = 12; ^b^n = 10; ^c^n = 1; ^d^n = 1

Results were expressed as estimated marginal means (± standard error), together with adjusted mean differences (Δ), 95% confidence intervals (95% CI), and p-values. Effect size was estimated using partial eta-squared (${\eta }_{p}^{2}$) and interpreted according to conventional cutoffs: 0.01 (small), 0.06 (medium), and 0.14 (large). Multiple comparisons between categories were adjusted using the Bonferroni correction. A significance level of 5% (*p* < 0.05) was adopted.

### Ethical aspects

This study was conducted in accordance with the Declaration of Helsinki. The Research Ethics Committee of the Federal University of Grande Dourados (UFGD) approved this study under protocol number 6.874.186. All patients provided written informed consent before study enrollment.

## Results

### Sample characteristics

**Fig. 1:**
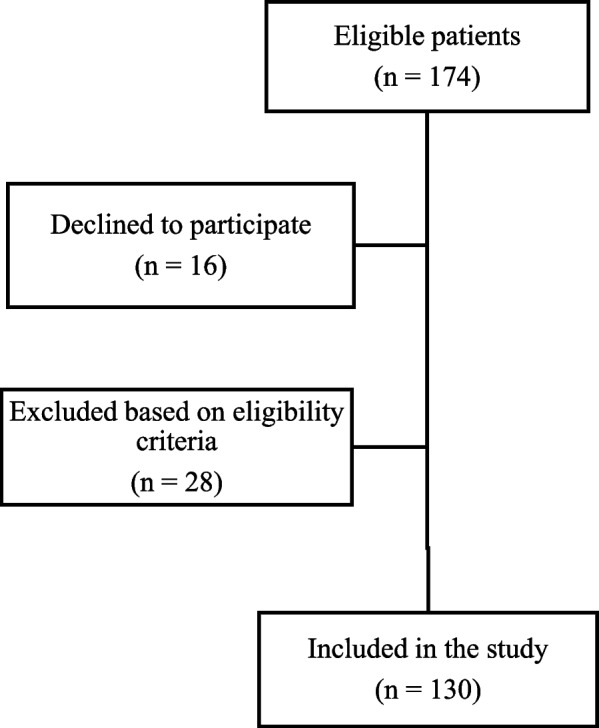
Flowchart of participant inclusion

Of the 174 eligible patients, 16 declined to participate, and 28 were excluded for pregnancy (n = 1) or inability to perform the physical test (n = 27). The final analytic sample comprised 130 patients (Fig. 1).

Mean age was 59.5 ± 11.6 years, and 73.8% were women (Table 1). Regarding self-reported race/skin color, 58.2% identified as Black or Brown. Just over half were married (56.2%), and 47.3% had more than 11 years of schooling. Socioeconomic class C was the most frequent (49.2%). In terms of lifestyle, 48.5% reported never drinking alcohol, 68.5% had never smoked, and 90.0% had an insufficient physical activity level.

For clinical characteristics, the most frequent cancer site was breast (53.1%), followed by the digestive system (18.5%) and prostate (11.5%). Overall, 39.8% of patients were in stage III disease, and 50.8% had metastases. Chemotherapy alone was the most common treatment modality (71.9%), and 19.8% received combined regimens involving two or more modalities (Table 1).

**Fig. 2:**
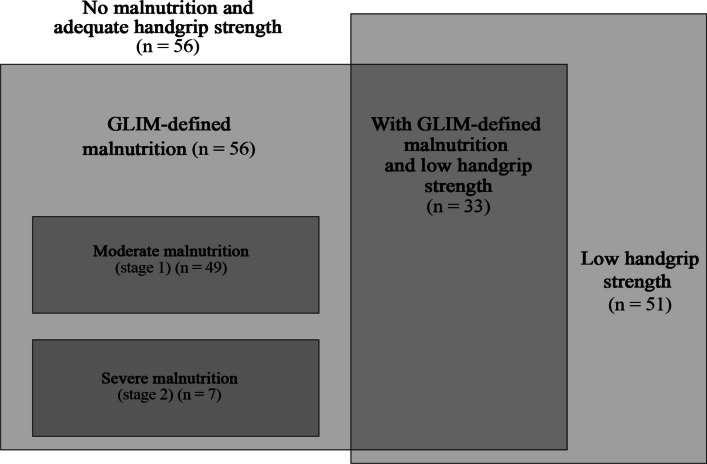
Distribution of participants by nutritional status and handgrip strength, highlighting moderate and severe GLIM-defined malnutrition, low handgrip strength, and their coexistence

Mean BMI was 27.2 ± 5.8 kg/m^2^, and excess body weight was observed in 66.4% of the sample. Low handgrip strength was present in 39.2% of patients, and GLIM-defined malnutrition in 43.1%. Regarding coexisting conditions, 25.4% had coexisting GLIM-defined malnutrition and low handgrip strength, whereas 43.1% had neither condition; 13.8% had low handgrip strength only, and 17.7% had GLIM-defined malnutrition only (Fig. 2; Supplementary Table 1).

Patients with GLIM-defined malnutrition had higher scores for fatigue, nausea and vomiting, and appetite loss. Those with low handgrip strength showed a broader pattern of impairment, with poorer scores on several functional scales, including role functioning (60.0 vs. 93.3; *p* < 0.001), and higher scores for fatigue, nausea and vomiting, and pain. Full results are presented in Supplementary Table 1.

The group with coexisting GLIM-defined malnutrition and low handgrip strength showed the worst HRQoL profile among all groups. Patients in the coexistence group had lower physical functioning scores (60.0 vs. 93.3 in the group with neither condition; r = 0.43; *p* < 0.001) and role functioning scores (58.3 vs. 100; r = 0.37; *p* < 0.001), as well as higher scores for fatigue (38.9 vs. 11.1; r = 0.41; *p* < 0.001) and nausea and vomiting (25.0 vs. 0.0; r = 0.38; *p* = 0.001). These findings indicate a consistent pattern of poorer HRQoL associated with coexisting GLIM-defined malnutrition and low handgrip strength. Complete comparisons are shown in Table 2 and Supplementary Table S2.

**Table 2:** Pairwise comparisons that remained significant after Bonferroni correction for the EORTC QLQ-C30 scales

Scales	Comparison	Z	p	r
Physical functioning	Neither condition × Low handgrip strength	−2.94	**0.003**	0.34
	Neither condition × Coexistence	−4.05	** < 0.001**	0.43
	Low handgrip strength × Malnutrition	−2.68	**0.007**	0.42
	Malnutrition × Coexistence	−3.19	**0.001**	0.38
	Neither condition × Low handgrip strength	−3.98	** < 0.001**	0.46
Role functioning	Neither condition × Coexistence	−3.51	** < 0.001**	0.37
	Low handgrip strength × Malnutrition	−3.68	** < 0.001**	0.57
	Malnutrition × Coexistence	−3.11	**0.002**	0.33
Emotional functioning	Neither condition × Coexistence	−2.28	**0.023**	0.24
	Malnutrition × Coexistence	−2.29	**0.022**	0.24
Fatigue	Neither condition × Low handgrip strength	−2.68	**0.007**	0.28
	Neither condition × Coexistence	−3.88	** < 0.001**	0.41
	Malnutrition × Coexistence	−3.05	**0.002**	0.39
Nausea and vomiting	Neither condition × Low handgrip strength	−2.27	**0.023**	0.25
	Neither condition × Coexistence	−3.42	**0.001**	0.38
	Malnutrition × Coexistence	−2.12	**0.034**	0.28
Pain	Neither condition × Coexistence	−2.76	**0.006**	0.29
	Malnutrition × Coexistence	−2.07	**0.039**	0.27
Dyspnea	Neither condition × Malnutrition	−2.27	**0.023**	0.25
	Low handgrip strength × Malnutrition	−2.66	**0.008**	0.38
	Malnutrition × Coexistence	−2.88	**0.004**	0.42
Appetite loss	Neither condition × Coexistence	−3.59	** < 0.001**	0.38
Diarrhea	Neither condition × Low handgrip strength	−2.08	**0.038**	0.24
	Neither condition × Coexistence	−2.26	**0.024**	0.24

Bold = statistically significant values. U: Mann–Whitney U statistic; Z: standardized Z-score; r: effect size

In univariate models, low handgrip strength was significantly associated with poorer physical, role, and emotional functioning, along with higher scores for fatigue, nausea and vomiting, pain, dyspnea, appetite loss, and diarrhea. GLIM-defined malnutrition alone was significantly associated only with appetite loss. After adjustment, low handgrip strength remained independently associated with all functional scales and with most symptom scales, whereas GLIM-defined malnutrition remained associated only with appetite loss. Full results, including Δ and ${\eta }_{p}^{2}$ values, are presented in Table 3.

**Table 3:** Univariate and adjusted models for the association between malnutrition, handgrip strength, and the EORTC QLQ-C30 scales

**Scales**	**Malnutrition (Δ)**	**p**	${{\boldsymbol{\eta}}}_{{\boldsymbol{p}}}^{2}$	**Handgrip strength (Δ)**	**p**	${{\boldsymbol{\eta}}}_{{\boldsymbol{p}}}^{2}$
**Univariate (unadjusted) models**						
Physical functioning	− 0.67	0.887	0.000	− 22.00	** < 0.001**	0.149
Role functioning	+ 9.02	0.158	0.016	− 31.04	** < 0.001**	0.160
Emotional functioning	+ 1.37	0.816	0.000	− 18.70	**0.002**	0.075
Fatigue	+ 5.05	0.370	0.006	+ 24.40	** < 0.001**	0.130
Nausea and vomiting	+ 4.49	0.350	0.007	+ 14.80	**0.002**	0.070
Pain	+ 8.51	0.176	0.014	+ 13.54	**0.032**	0.036
Dyspnea	+ 4.80	0.242	0.011	+ 10.01	**0.016**	0.045
Appetite loss	+ 13.04	**0.042**	0.033	+ 16.42	**0.011**	0.051
Diarrhea	− 1.01	0.845	0.000	+ 10.67	**0.040**	0.034
**Adjusted models**						
Physical functioning	−13.52	0.079	0.045	−27.57	** < 0.001**	0.231
Role functioning	−11.68	0.264	0.019	−35.84	** < 0.001**	0.214
Emotional functioning	−7.71	0.381	0.011	−21.17	**0.004**	0.118
Fatigue	+ 14.59	0.118	0.036	+ 29.32	** < 0.001**	0.187
Nausea and vomiting	+ 11.09	0.125	0.035	+ 24.27	** < 0.001**	0.208
Pain	+ 18.57	0.068	0.049	+ 23.15	**0.006**	0.108
Dyspnea	+ 6.49	0.252	0.020	+ 14.03	**0.003**	0.124
Appetite loss	+ 23.29	**0.027**	0.071	+ 22.86	**0.009**	0.099
Diarrhea	−2.19	0.795	0.001	+ 8.46	0.222	0.023

Bold = statistically significant values. Δ values represent mean differences between groups (yes vs. no for malnutrition; low vs. adequate for handgrip strength).${\eta }_{p}^{2}$= partial eta-squared. All models were adjusted for sex, age, tumor site, clinical stage, and body mass index

In univariate models, coexisting GLIM-defined malnutrition and low handgrip strength were associated with poorer physical and role functioning and higher levels of fatigue, nausea and vomiting, pain, and appetite loss compared with either condition alone. Additional differences between GLIM-defined malnutrition alone and coexistence were particularly evident for physical and role functioning and fatigue. After model adjustment, coexistence remained associated with poorer physical, role, and emotional functioning, as well as higher scores for fatigue, nausea and vomiting, pain, dyspnea, and appetite loss, indicating an independent impact of coexisting GLIM-defined malnutrition and low handgrip strength on HRQoL. Detailed results are shown in Table 4.

**Table 4:** Univariate and adjusted models for the association between coexistence and the EORTC QLQ-C30 scales

**Scales**	**Neither condition** **Mean (95% CI)**	**Malnutrition** **Mean (95% CI)**	**Low handgrip strength** **Mean (95% CI)**	**Coexistence** **Mean (95% CI)**	**Significant Δ (I–J)**	***p*-value**	${{\boldsymbol{\eta}}}_{{\boldsymbol{p}}}^{2}$	***p* (model)**
**Univariate (unadjusted) models**								
Physical functioning	82.3 (75.8–88.7)	83.2 (73.1–93.2)	61.9 (50.5–73.2)	59.6 (51.2–68.0)	Neither condition – Low handgrip strength (−20.4)	**0.015**	0.169	** < 0.001**
					Neither condition – Coexistence (−22.7)	** < 0.001**		
					Low handgrip strength – Malnutrition (+ 21.3)	**0.037**		
					Malnutrition – Coexistence (−23.6)	**0.003**		
Role functioning	83.6 (74.9–92.4)	89.1 (75.5–102.8)	49.1 (33.6–64.50	61.6(50.2–73.0)	Neither condition – Low handgrip strength (−34.6)	**0.001**	0.162	** < 0.001**
					Neither condition – Coexistence (−22.0)	**0.018**		
					Low handgrip strength – Malnutrition (+ 40.1)	**0.001**		
					Malnutrition – Coexistence (−27.5)	**0.016**		
Fatigue	20.0 (12.3–27.8)	20.8 (8.7–32.9)	40.1 (26.5–53.8)	49.5 (39.4–59.6)	Neither condition – Coexistence (+ 29.5)	** < 0.001**	0.168	** < 0.001**
					Malnutrition – Coexistence (+ 28.7)	**0.003**		
Nausea and vomiting	11.0 (4.4–17.6)	13.8 (3.5–24.1)	24.1 (12.4–35.7)	30.3 (21.7–38.9)	Neither condition – Coexistence (+ 19.3)	**0.004**	0.101	**0.004**
Pain	17.9 (9.2–26.5)	18.1 (4.7–31.6)	23.1 (7.9–38.3)	39.9 (28.7–51.1)	Neither condition – Coexistence (+ 22.0)	**0.015**	0.078	**0.017**
Appetite loss	10.1 (1.4–18.8)	18.8 (5.2–32.4)	22.2 (6.8–37.6)	39.6 (28.0–51.1)	Neither condition – Coexistence (+ 29.5)	**0.001**	0.116	**0.001**
**Adjusted models**								
Physical functioning	83.4 (73.5–93.3)	82.1 (69.0–95.2)	67.3 (51.8–82.8)	48.3 (34.1–62.5)	Neither condition – Coexistence (−35.1)	**0.002**	0.240	** < 0.001**
					Malnutrition – Coexistence (−33.8)	**0.001**		
Role functioning	85.2 (71.7–98.6)	86.4 (68.5–104.3)	58.3 (37.0–79.5)	44.9 (25.5–64.4)	Neither condition – Coexistence (−40.3)	**0.014**	0.216	**0.001**
					Malnutrition – Coexistence (−41.6)	**0.005**		
Emotional functioning	72.5 (61.0–83.9)	75.2 (60.1–90.4)	60.9 (43.0–78.8)	47.5 (31.1–63.9)	Malnutrition – Coexistence (−27.7)	**0.044**	0.125	**0.029**
Fatigue	19.5 (7.5–31.6)	19.4 (3.5–35.3)	34.1 (15.2–53.0)	57.4 (40.1–74.6)	Neither condition – Coexistence (+ 37.9)	**0.008**	0.201	**0.002**
					Malnutrition – Coexistence (+ 38.0)	**0.004**		
Nausea and vomiting	6.8 (−2.5–16.1)	11.4 (−0.9–23.8)	27.3 (12.6–41.9)	36.3 (22.9–49.7)	Neither condition – Coexistence (+ 29.5)	**0.007**	0.209	**0.001**
					Malnutrition – Coexistence (+ 24.9)	**0.020**		
Pain	13.5 (0.4–26.6)	18.5 (1.2–35.9)	20.3 (−0.3–40.8)	49.5 (30.7–68.3)	Neither condition – Coexistence (+ 36.0)	**0.027**	0.134	**0.021**
Dyspnea	4.8 (−2.5–12.1)	4.5 (−5.2–14.2)	12.3 (0.8–23.9)	22.5(11.9–33.0)	Malnutrition – Coexistence (+ 18.0)	**0.042**	0.132	**0.023**
Appetite loss	6.1 (−7.5–19.7)	22.1 (3.9–40.2)	19.7 (−1.6–41.0)	45.7 (26.4–64.9)	Neither condition – Coexistence (+ 39.6)	**0.015**	0.132	**0.022**

Bold = statistically significant values. Mean = estimated marginal means.${\eta }_{p}^{2}$= partial eta-squared. Significant Δ (I–J) between groups – multiple comparisons with Bonferroni correction. All models were adjusted for sex, age, tumor site, clinical stage, and body mass index

## Discussion

Our study showed that coexisting GLIM-defined malnutrition and low handgrip strength were associated with poorer health-related quality of life (HRQoL) in patients with cancer. However, low handgrip strength alone was already a key marker, yielding HRQoL scores as unfavorable as those observed in the coexistence group, corroborating evidence that handgrip strength (HGS) is an independent prognostic marker in oncology [32, 33]. The analysis of estimated marginal means, therefore, suggests that coexisting GLIM-defined malnutrition and low handgrip strength are associated with functional impairment. In the adjusted models, this combined condition was associated with poorer physical, role, and emotional functioning, as well as higher levels of fatigue, nausea and vomiting, pain, dyspnea, and appetite loss, underscoring the central role of muscle strength in HRQoL impairment as captured by the EORTC QLQ-C30.

These findings are consistent with recent literature showing the relevance of both GLIM-defined malnutrition and low handgrip strength in determining HRQoL among patients with cancer. Landgrebe et al*.* [9] observed that GLIM-defined malnutrition was associated with poorer HRQoL, whereas Caroline et al*.* [11] and Silva et al*.* [10] reported that low handgrip strength is an independent marker of worse functional prognosis. Similarly, Kim et al*.* [34] found that decreased HGS in women was associated with poorer HRQoL. Taken together, these findings support our results and highlight the importance of integrating nutritional and functional indicators into the clinical assessment of this population.

The stronger association of low handgrip strength with poorer HRQoL, even in the absence of GLIM-defined malnutrition, may be explained by HGS directly reflecting patients’ functional capacity. As physical and role functioning scores decline, autonomy in daily activities decreases, which in turn contributes to psychological distress and a poorer perception of HRQoL. As previously emphasized by Rier et al*.* [5] and Nipp et al*.* [35], muscle function is a key component of independence—a central element of the HRQoL experience for patients with cancer. Importantly, reduced HGS may also reflect broader disease burden or treatment-related effects that cannot be fully disentangled in a cross-sectional design. Given the high prevalence of excess body weight in our sample, functional measures such as HGS may be particularly informative, as unintentional weight loss remains clinically meaningful even among patients with elevated BMI and adverse body composition changes may be masked.

In addition to the functional scales, greater intensity of dyspnea, pain, fatigue, and gastrointestinal symptoms such as nausea, vomiting, and appetite loss were also associated with poorer HRQoL, in line with the findings of Anandavadivelan et al*.* [36] and Galindo et al*.* [37]. These results suggest that the interplay between functional decline (low handgrip strength) and nutritional disturbances (greater gastrointestinal symptom burden) is linked to a poorer overall condition in patients with cancer.

To date, no studies have evaluated the impact of coexisting GLIM-defined malnutrition and low handgrip strength on HRQoL in patients with cancer. Although each condition has been independently associated with poorer HRQoL, our findings suggest that their coexistence is linked to more severe impairment, particularly in physical and role functioning, as well as a greater burden of fatigue, gastrointestinal symptoms, and pain. These results indicate an additive burden when malnutrition and low muscle strength occur together, leading to worse HRQoL outcomes than when either condition is present alone. To our knowledge, this is a novel observation and underscores the importance of considering nutritional and functional indicators together when interpreting HRQoL in patients with cancer.

From a clinical standpoint, these findings reinforce the need to integrate early nutritional diagnosis and routine assessment of muscle strength into oncology and nutrition care. Multiprofessional strategies such as nutritional support, symptom management for problems that limit food intake, and structured exercise programs may help preserve muscle function and, consequently, attenuate HRQoL decline.

### Strengths and limitations

Strengths of this study include the use of the GLIM criteria, currently recommended for the diagnosis of malnutrition, and the concurrent assessment of handgrip strength, a functional marker with well-established prognostic value. The combined analysis of these indicators in a sample of Brazilian patients with cancer adds new evidence, broadens the geographic diversity of available data, and provides a basis for more integrated multiprofessional practice.

This study also has limitations. The cross-sectional design precludes causal inference, and the sample was heterogeneous, including patients with different tumor types, clinical stages, and treatment modalities. In addition, the predominance of women and patients with breast cancer in our sample may limit the generalisability of these findings to other oncology populations. Although these factors may influence the observed associations, our models were adjusted for key clinical variables, mitigating this concern. In addition, according to the GLIM criteria, cancer was considered an etiologic criterion given its frequent association with systemic inflammation and metabolic stress. However, inflammatory burden may vary across tumor types and disease stages, and the absence of objective inflammatory biomarkers (e.g., C-reactive protein) limited a more precise characterization of inflammation in our study population. Although the GLIM approach recommends an initial nutritional risk screening step, we applied the diagnostic assessment directly given the high-risk profile of patients in tertiary oncology care. Future studies may further examine whether prior screening influences prevalence estimates and associations with clinical outcomes.

Another limitation is the use of univariate general linear models, which do not examine multiple outcomes simultaneously and may fail to capture more complex interdependencies; future studies using multivariate approaches could complement our findings. In addition, the large number of comparisons across EORTC QLQ-C30 scales required Bonferroni correction, which is known to be conservative, particularly when tests are highly correlated. Under these circumstances, some clinically relevant associations may not have reached statistical significance because of type II error, given that the sample size, although adequate overall, may not have provided ≥ 80% power for all analyses.

Despite these limitations, this study is innovative in jointly examining coexisting GLIM-defined malnutrition and low handgrip strength, providing relevant evidence on factors that contribute to greater clinical vulnerability and poorer health-related quality of life in patients with cancer.

## Conclusion

In patients with cancer, coexisting GLIM-defined malnutrition and low handgrip strength are associated with greater impairment in health-related quality of life, particularly on functional and symptom scales. Low handgrip strength showed a particularly relevant association with poorer HRQoL outcomes, even in the absence of GLIM-defined malnutrition. These findings highlight the importance of jointly assessing nutritional and functional indicators to identify patients at higher risk of vulnerability and impaired HRQoL. Early multiprofessional interventions aimed at preserving muscle function and managing malnutrition may help maintain HRQoL and should be considered in oncology care.

## Supplementary Information

Supplementary Material 1.

## Data Availability

All data supporting the findings of this study can be obtained from the corresponding author upon request.

## Abbreviations

AP: Appetite Loss
DY: Dyspnoea
EF: Emotional Functioning
EORTC QLQ-C30: European Organisation for Research and Treatment of Cancer Quality of Life Questionnaire Core 30
FA: Fatigue
GLIM: Global Leadership Initiative on Malnutrition
GLM-Univariate: General Linear Models - Univariate
HGS: Handgrip Strength
HRQoL: Health-Related Quality of Life
IMC: Body Mass Index
NV: Nausea and Vomiting
PA: Pain
PF: Physical Functioning
RF: Role Functioning
