# Supplementary material for: Low handgrip strength, GLIM-defined malnutrition, or their coexistence: which has the greatest impact on health-related quality of life in patients with cancer?
Source: BMC Cancer. 2026 Mar 21;26:545. doi: 10.1186/s12885-026-15823-8 (PMC13134090; doi:10.1186/s12885-026-15823-8)
Supplement: Supplementary file 1 — Supplementary Material 1. [file 12885_2026_15823_MOESM1_ESM.docx]

**Supplementary material**

**Supplementary Table S1** – Distribution of EORTC QLQ-C30 scale scores according to GLIM-defined malnutrition, handgrip strength, and the coexistence of GLIM-defined malnutrition and low handgrip strength

| **EORTC Score** | **GLIM-defined malnutrition**  **n = 130** | | | | **p-value** | **Handgrip strength**  **n = 130** | | | | **p-value** | **Coexistence**  **n = 130** | | | | | | | | **p-value** |
| --- | --- | --- | --- | --- | --- | --- | --- | --- | --- | --- | --- | --- | --- | --- | --- | --- | --- | --- | --- |
|  | **No**  **n = 74** | | **Yes**  **n = 56** | |  | **Adequate**  **n = 79** | | **Low handgrip strength**  **n = 51** | |  | **Neither condition**  **n = 56** | | **Low handgrip strength**  **n = 18** | | **Malnutrition**  **n = 23** | | **Both conditions**  **n = 33** | |  |
|  | Mdn | IQR | Mdn | IQR |  | Mdn | IQR | Mdn | IQR |  | Mdn | IQR | Mdn | IQR | Mdn | IQR | Mdn | IQR |  |
| **PF** | 86.6 | 60.0-100 | 76.6 | 48.3-93.3 | 0.074 | 93.3 | 73.3-100 | 60.0 | 40.0-86.7 | **<0.001** | 93.3 | 73.3-93.3 | 56.7 | 40.0-86.7 | 93.3 | 73.3-100 | 60.0 | 36.7-83.3 | **<0.001** |
| **RF** | 100 | 66.6-100 | 83.3 | 66.6-100 | 0.369 | 100 | 83.3-100 | 66.7 | 16.7-100 | **<0.001** | 100 | 83.3-100 | 58.3 | 0.0-83.3 | 100 | 83.3-100 | 66.7 | 25.0-100 | **<0.001** |
| **EF** | 75.0 | 41.6-100 | 75.0 | 35.4-91.6 | 0.428 | 83.3 | 50.0-100 | 58.3 | 25.0-83.3 | **0.003** | 79.2 | 50.0-100 | 58.3 | 22.9-85.4 | 83.3 | 58.3-100 | 58.3 | 25.0-83.3 | **0.029** |
| **CF** | 83.3 | 66.6-100 | 75.0 | 50.0-100 | 0.184 | 83.3 | 66.7-100 | 66.7 | 50.0-100 | **0.024** | 83.3 | 66.7-100 | 83.3 | 50.0-100 | 83.3 | 66.7-100 | 66.7 | 50.0-91.7 | 0.130 |
| **SF** | 100 | 62.5-100 | 83.3 | 66.6-100 | 0.859 | 100 | 66.7-100 | 83.3 | 50.0-100 | 0.149 | 100 | 66.7-100 | 75.0 | 50.0-100 | 100 | 66.7-100 | 83.3 | 66.7-100 | 0.511 |
| **FA** | 11.1 | 0.0-44.4 | 33.3 | 2.7-66.6 | **0.018** | 11.1 | 0.0-33.3 | 44.4 | 11.1-77.8 | **<0.001** | 11.1 | 0.0-30.5 | 38.9 | 11.1-69.4 | 22.2 | 0.0-33.3 | 44.4 | 16.7-77.8 | **<0.001** |
| **NV** | 0.0 | 0.0-33.3 | 16.6 | 0.0-45.8 | **0.036** | 0.0 | 0.0-16.7 | 16.7 | 0.0-50.0 | **<0.001** | 0.0 | 0.0-16.7 | 25.0 | 0.0-50.0 | 0.0 | 0.0-16.7 | 16.7 | 0.0-50.0 | **0.003** |
| **PA** | 0.0 | 0.0-33.3 | 16.6 | 0.0-62.5 | 0.052 | 0.0 | 0.0-33.3 | 16.7 | 0.0-66.7 | **0.016** | 0.0 | 0.0-33.3 | 0.0 | 0.0-33.3 | 0.0 | 0.0-16.7 | 33.3 | 0.0-83.3 | **0.038** |
| **DY** | 0.0 | 0.0-0.0 | 0.0 | 0.0-0 | 0.592 | 0.0 | 0.0-0.0 | 0.0 | 0.0-33.3 | **0.030** | 0.0 | 0.0-0.0 | 0.0 | 0.0-33.3 | 0.0 | 0.0-0.0 | 0.0 | 0.0-33.3 | **0.036** |
| **SL** | 0.0 | 0.0-66.6 | 0.0 | 0.0-66.6 | 0.857 | 0.0 | 0.0-33.3 | 33.3 | 0.0-66.7 | **0.014** | 0.0 | 0.0-33.3 | 33.3 | 0.0-75.0 | 0.0 | 0.0-33.3 | 0.0 | 0.0-83.3 | 0.085 |
| **AP** | 0.0 | 0.0-0.0 | 0.0 | 0.0-66.6 | **0.005** | 0.0 | 0.0-0.0 | 0.0 | 0.0-66.7 | **0.002** | 0.0 | 0.0-0.0 | 0.0 | 0.0-41.7 | 0.0 | 0.0-33.3 | 16.7 | 0.0-100 | **0.004** |
| **CO** | 0.0 | 0.0-33.3 | 0.0 | 0.0-33.3 | 0.641 | 0.0 | 0.0-0.0 | 0.0 | 0.0-66.7 | **0.036** | 0.0 | 0.0-0.0 | 0.0 | 0.0-75.0 | 0.0 | 0.0-0.0 | 0.0 | 0.0-66.7 | 0.187 |
| **DI** | 0.0 | 0.0-0.0 | 0.0 | 0.0-33.3 | 0.424 | 0.0 | 0.0-0.0 | 0.0 | 0.0-33.3 | **0.004** | 0.0 | 0.0-0.0 | 0.0 | 0.0-33.3 | 0.0 | 0.0-0.0 | 0.0 | 0.0-33.3 | **0.039** |
| **FI** | 0.0 | 0.0-66.6 | 33.3 | 0.0-66.68 | 0.515 | 0.0 | 0.0-33.3 | 0.0 | 0.0-66.7 | 0.651 | 0.0 | 0.0-66.7 | 0.0 | 0.0-66.7 | 0.0 | 0.0-33.3 | 33.3 | 0.0-66.7 | 0.564 |
| **GHS** | 83.3 | 66.6-93.7 | 83.3 | 66.6-100 | 0.462 | 83.3 | 66.7-100 | 83.3 | 58.3-91.7 | 0.651 | 83.3 | 66.7-100 | 83.3 | 56.2-91.7 | 83.3 | 75.0-100 | 83.3 | 66.7-100 | 0.773 |

**Legend:** Mdn = median; IQR = interquartile range; PF = physical functioning; RF = role functioning; EF = emotional functioning; CF = cognitive functioning; SF = social functioning; FA = fatigue; NV = nausea and vomiting; PA = pain; DY = dyspnea; SL = insomnia; AP = appetite loss; CO = constipation; DI = diarrhea; FI = financial difficulties; GHS = global health status. Bold = statistically significant values.

**Supplementary Table S2** – Pairwise comparisons between groups (neither condition, low handgrip strength only, malnutrition only, and coexistence) for all EORTC QLQ-C30 scales

| **Scale** | **Comparison** | **U** | **Z** | **p** | **r** |
| --- | --- | --- | --- | --- | --- |
| PF | Neither condition × Low handgrip strength | 274.5 | -2.94 | **0.003** | 0.34 |
|  | Neither condition × Malnutrition | 641.0 | -0.03 | 0.973 | 0.00 |
|  | Neither condition × Coexistence | 452.0 | -4.05 | **<0.001** | 0.43 |
|  | Low handgrip strength × Malnutrition | 106.5 | -2.68 | **0.007** | 0.42 |
|  | Low handgrip strength × Coexistence | 290.0 | -0.14 | 0.890 | 0.02 |
|  | Malnutrition × Coexistence | 189.0 | -3.19 | **0.001** | 0.38 |
| RF | Neither condition × Low handgrip strength | 221.5 | -3.98 | **<0.001** | 0.46 |
|  | Neither condition × Malnutrition | 634.5 | -0.13 | 0.898 | 0.01 |
|  | Neither condition × Coexistence | 550.5 | -3.51 | **<0.001** | 0.37 |
|  | Low handgrip strength × Malnutrition | 74.5 | -3.68 | **<0.001** | 0.57 |
|  | Low handgrip strength × Coexistence | 241.0 | -1.13 | 0.260 | 0.16 |
|  | Malnutrition × Coexistence | 203.0 | -3.11 | **0.002** | 0.33 |
| EF | Neither condition × Low handgrip strength | 361.5 | -1.82 | 0.069 | 0.21 |
|  | Neither condition × Malnutrition | 612.5 | -0.35 | 0.729 | 0.04 |
|  | Neither condition × Coexistence | 659.0 | -2.28 | **0.023** | 0.24 |
|  | Low handgrip strength × Malnutrition | 133.5 | -1.95 | 0.051 | 0.24 |
|  | Low handgrip strength × Coexistence | 290.0 | -0.14 | 0.890 | 0.02 |
|  | Malnutrition × Coexistence | 243.5 | -2.29 | **0.022** | 0.24 |
| FA | Neither condition × Low handgrip strength | 299.0 | -2.68 | **0.007** | 0.28 |
|  | Neither condition × Malnutrition | 563.5 | -0.91 | 0.362 | 0.10 |
|  | Neither condition × Coexistence | 480.5 | -3.88 | **<0.001** | 0.41 |
|  | Low handgrip strength × Malnutrition | 131.0 | -2.03 | **0.043** | 0.32 |
|  | Low handgrip strength × Coexistence | 254.0 | -0.85 | 0.393 | 0.12 |
|  | Malnutrition × Coexistence | 198.5 | -3.05 | **0.002** | 0.39 |
| NV | Neither condition × Low handgrip strength | 350.5 | -2.27 | **0.023** | 0.25 |
|  | Neither condition × Malnutrition | 582.0 | -0.81 | 0.416 | 0.09 |
|  | Neither condition × Coexistence | 565.0 | -3.42 | **0.001** | 0.38 |
|  | Low handgrip strength × Malnutrition | 159.0 | -1.38 | 0.169 | 0.22 |
|  | Low handgrip strength × Coexistence | 268.5 | -0.58 | 0.560 | 0.08 |
|  | Malnutrition × Coexistence | 259.5 | -2.12 | **0.034** | 0.28 |
| PA | Neither condition × Low handgrip strength | 471.0 | -0.47 | 0.636 | 0.05 |
|  | Neither condition × Malnutrition | 626.0 | -0.22 | 0.825 | 0.02 |
|  | Neither condition × Coexistence | 625.0 | -2.76 | **0.006** | 0.29 |
|  | Low handgrip strength × Malnutrition | 196.0 | -0.32 | 0.749 | 0.05 |
|  | Low handgrip strength × Coexistence | 223.5 | -1.52 | 0.129 | 0.21 |
|  | Malnutrition × Coexistence | 261.5 | -2.07 | **0.039** | 0.27 |
| DY | Neither condition × Low handgrip strength | 459.0 | -0.79 | 0.430 | 0.09 |
|  | Neither condition × Malnutrition | 517.5 | -2.27 | **0.023** | 0.25 |
|  | Neither condition × Coexistence | 824.0 | -1.15 | 0.252 | 0.12 |
|  | Low handgrip strength × Malnutrition | 149.5 | -2.66 | **0.008** | 0.38 |
|  | Low handgrip strength × Coexistence | 292.5 | -0.11 | 0.912 | 0.02 |
|  | Malnutrition × Coexistence | 264.5 | -2.88 | **0.004** | 0.42 |
| AP | Neither condition × Low handgrip strength | 393.0 | -1.95 | 0.052 | 0.23 |
|  | Neither condition × Malnutrition | 530.5 | -1.71 | 0.088 | 0.19 |
|  | Neither condition × Coexistence | 568.0 | -3.59 | **<0.001** | 0.38 |
|  | Low handgrip strength × Malnutrition | 196.5 | -0.32 | 0.748 | 0.05 |
|  | Low handgrip strength × Coexistence | 232.0 | -1.24 | 0.214 | 0.18 |
|  | Malnutrition × Coexistence | 282.0 | -1.63 | 0.103 | 0.22 |
| DI | Neither condition × Low handgrip strength | 380.5 | -2.08 | **0.038** | 0.24 |
|  | Neither condition × Malnutrition | 624.0 | -0.15 | 0.878 | 0.02 |
|  | Neither condition × Coexistence | 691.0 | -2.26 | **0.024** | 0.24 |
|  | Low handgrip strength × Malnutrition | 156.5 | -1.76 | 0.078 | 0.28 |
|  | Low handgrip strength × Coexistence | 281.0 | -0.16 | 0.870 | 0.02 |
|  | Malnutrition × Coexistence | 284.0 | -1.83 | 0.067 | 0.25 |

**Legend:** PF = physical functioning; RF = role functioning; EF = emotional functioning; FA = fatigue; NV = nausea and vomiting; PA = pain; DY = dyspnea; AP = appetite loss; DI = diarrhea. Bold = statistically significant values. U: Mann–Whitney U statistic; Z: standardized Z-score; r: effect size. p-values in bold indicate statistical significance (p < 0.05).


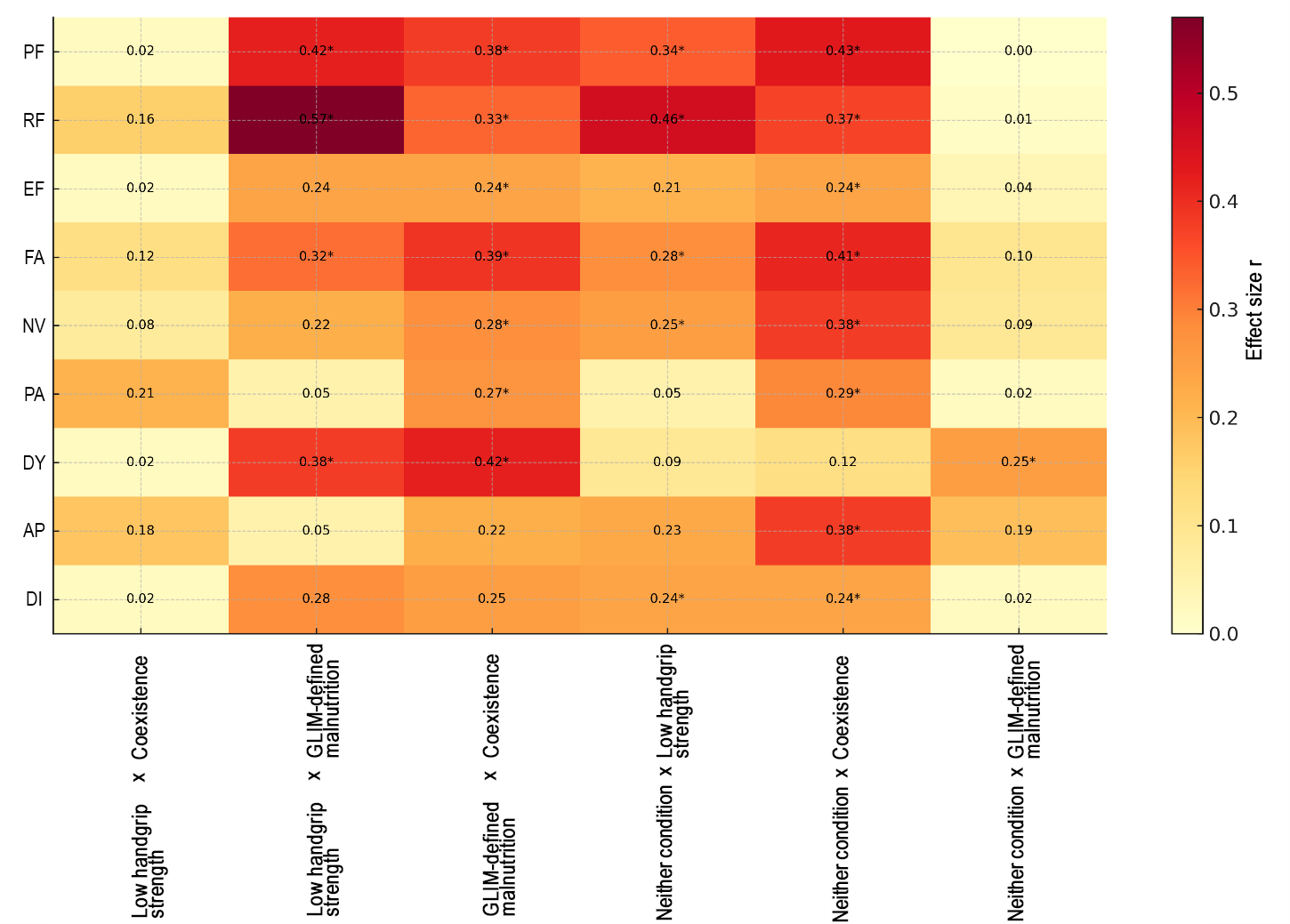
**Supplementary figure S1**

**Heat map of effect sizes (r) for the EORTC QLQ-C30 scales.** Legend: PF = physical functioning; RF = role functioning; EF = emotional functioning; FA = fatigue; NV = nausea and vomiting; PA = pain; DY = dyspnea; AP = appetite loss; DI = diarrhea. The values inside the cells represent r, and an asterisk (*) indicates statistical significance (p < 0.05).
